# Association of *ERBB2* gene status with histopathological parameters and disease-specific survival in gastric carcinoma patients

**DOI:** 10.1038/sj.bjc.6604885

**Published:** 2009-01-20

**Authors:** J D Barros-Silva, D Leitão, L Afonso, J Vieira, M Dinis-Ribeiro, M Fragoso, M J Bento, L Santos, P Ferreira, S Rêgo, C Brandão, F Carneiro, C Lopes, F Schmitt, M R Teixeira

**Affiliations:** 1 Department of Genetics, Portuguese Oncology Institute, Porto, Portugal; 2 Institute of Molecular Pathology and Immunology of Porto University (IPATIMUP), Porto, Portugal; 3 Department of Pathology, Portuguese Oncology Institute, Porto, Portugal; 4 Department of Gastroenterology, Portuguese Oncology Institute, Porto, Portugal; 5 Department of Oncology, Portuguese Oncology Institute, Porto, Portugal; 6 Department of Epidemiology, Portuguese Oncology Institute, Porto, Portugal; 7 Department of Surgery, Portuguese Oncology Institute, Porto, Portugal; 8 Abel Salazar Biomedical Sciences Institute, University of Porto, Porto, Portugal

**Keywords:** gastric cancer, *ERBB2*, amplification, fluorescence *in situ* hybridisation

## Abstract

The clinical significance of *ERBB2* amplification/overexpression in gastric cancer remains unclear. In this study, we evaluated the ERBB2 status in 463 gastric carcinomas using immunohistochemistry (IHC) and fluorescence *in situ* hybridisation (FISH), and compared the findings with histopathological characteristics and with disease-specific survival. ERBB2 overexpression (2+ and 3+) and amplification (ratio *ERBB2*/CEP17⩾2) were found in 43 (9.3%) and 38 (8.2%) gastric carcinomas, respectively. Perfect IHC/FISH correlation was found for the 19 cases scored as 0 (all negative by FISH), and also for the 25 cases scored as 3+ (all positive by FISH). One out of six carcinomas scored as 1+ and 12 out of 18 carcinomas scored as 2+ were positive by FISH. *ERBB2* amplification was associated with gastric carcinomas of intestinal type (*P*=0.007) and with an expansive growth pattern (*P*=0.021). *ERBB2* amplification was detected in both histological components of two mixed carcinomas, indicating a common clonal origin. A statistically significant association was found between *ERBB2* amplification and worse survival in patients with expansive gastric carcinomas (*P*=0.011). We conclude that ERBB2 status may have clinical significance in subsets of gastric cancer patients, and that further studies are warranted to evaluate whether patients whose gastric carcinomas present *ERBB2* amplification/overexpression may benefit from therapy targeting this surface receptor.

Despite the trend for decreasing incidence, gastric adenocarcinoma is still the second cause of cancer death worldwide (Parkin *et al*, 2005). The overall 5-year survival rate of patients with resectable gastric cancer ranges from 10 to 30% (Harrison *et al*, 1998; Msika *et al*, 2000; Green *et al*, 2002). Apart from surgical resection, evaluation of available therapies, both neo-adjuvant and adjuvant, provides conflicting results regarding the clinical outcome. Several meta-analyses have been published in an attempt to address the discrepancies reported in the literature, but recommendation for adjuvant chemotherapy in Western centres is still not consensual (Hermans *et al*, 1993; Earle and Maroun, 1999; Mari *et al*, 2000; Gianni *et al*, 2001; Janunger *et al*, 2001, 2002; Hu *et al*, 2002). The most important prognostic factor established for gastric cancer is the TNM stage, which is determined by the depth of invasion, involvement of lymph nodes, and distant metastasis. However, clinical outcome varies among patients in the same stage (Park *et al*, 2006). Therefore prognostic factors other than the TNM stage, as well as new therapies, would be of great value for gastric cancer patients.

The *ERBB2* gene maps to 17q12–q21 and encodes a 185-kDa transmembrane tyrosine kinase receptor (p185), which is a member of the epidermal growth factor receptor family (Xu *et al*, 1984; Akiyama *et al*, 1986; Popescu *et al*, 1989). In breast carcinomas, *ERBB2* functions as an oncogene, as amplification of the gene induces protein overexpression in the cell membrane (Slamon *et al*, 1989). Besides being a poor prognosis marker (Dowsett *et al*, 2003), *ERBB2* amplification is a predictive marker for targeted therapy with the monoclonal antibody trastuzumab in breast cancer patients with metastatic disease (Slamon *et al*, 2001). More recently, trastuzumab was shown to be effective as adjuvant treatment in breast-carcinoma patients with *ERBB2* amplification/overexpression (Piccart-Gebhart *et al*, 2005; Romond *et al*, 2005).

Apart from breast cancer, *ERBB2* amplification has been found in other malignant tumours, such as ovarian (McKenzie *et al*, 1993), lung (Hirashima *et al*, 2001), colon (Cohen *et al*, 1989), and gastric carcinomas (David *et al*, 1992). Earlier studies in gastric carcinomas reported ERBB2 overexpression using immunohistochemistry (IHC) in 5.2–22.6% of the cases, whereas the proportion with *ERBB2* amplification evaluated using fluorescence *in situ* hybridisation (FISH) ranged from 3.8 to 12.2% (Takehana *et al*, 2002; Varis *et al*, 2004; Park *et al*, 2006; Kim *et al*, 2007). However, the clinical significance of *ERBB2* amplification/overexpression in gastric cancer patients is still not clear, because most studies had neither follow-up data nor statistical power for that purpose (David *et al*, 1992; Nakajima *et al*, 1999; Takehana *et al*, 2002; Kimura *et al*, 2004; Kim *et al*, 2007).

Recently, Tanner *et al* (2005) showed that the gastric-cancer cell line N87 presenting *ERBB2* amplification is as sensitive to trastuzumab as the *ERBB2*-amplified breast-carcinoma cell line SKBR-3, which is a widely used reference in trastuzumab sensitivity studies (Pegram *et al*, 1997, 1999). Furthermore, Rebischung *et al* (2005) reported a case of a gastric cancer patient with ERBB2 overexpression (3+) who responded to a combination of chemotherapy and trastuzumab. A large-scale clinical study is currently being carried out to compare the response to chemotherapy combined with trastuzumab *vs* chemotherapy alone in gastric carcinoma patients with *ERBB2* amplification.

To clarify the potential clinical relevance of ERBB2 status in gastric cancer, we have characterised its overexpression and amplification in 463 patients with clinicopathological data and a follow-up time of 6–10 years.

## Materials and methods

### Type of study and selection of participants

A two-step study design was used to select the gastric cancer patients. First, a cross-sectional study was used to select 463 consecutive primary gastric adenocarcinoma patients who underwent gastrectomy at the Portuguese Oncology Institute—Porto (between 1996 and 2000) to assess the frequency of *ERBB2* overexpression and amplification. Simultaneously, the first step selected patients with *ERBB2* amplification for the second step, resulting in a retrospective prognostic cohort study (follow-up time from 1 to 133 months, mean 52.8 months), which included all patients with *ERBB2* amplification (*n*=38) and randomly selected patients with no amplification (*n*=218).

The tissue specimens for IHC and FISH analyses were archival tumour samples of surgically resected gastric carcinomas from the 463 patients. Patient age at diagnosis ranged from 26 to 91 years (median, 67 years).

### Variables

Clinical data were collected by a group of clinicians blinded to ERBB2 status, using a datasheet specifically developed for this study, including the following parameters: age, gender, date of and status on last follow-up, surgery type (curative or palliative according to the surgeon) and date, TNM stage, and treatment other than surgery (if any). Time to clinical outcome was considered from the date of surgery until the last clinical appointment attended, and each patient was classified under one of the following categories: alive with no evidence of disease, alive with disease, dead with no evidence of disease, and dead from disease. For histological data collection, pathologists reviewed a representative H&E-stained slide.

### Immunohistochemistry

Immunohistochemistry targeting the ERBB2 protein was carried out in 4-*μ*m-thick tissue sections. The monoclonal antibody NCL-CB11 (mouse monoclonal antibody, Novocastra Laboratories Ltd, Newcastle upon Tyne, UK), recognising the intracellular portion of the protein, was used. Tissue sections were deparaffinised followed by antigen retrieval in citrate buffer (0.01 M, pH 6.0) at high temperature (water bath at 98 °C). After blocking for non-specific binding, the primary antibody was added in a pre-standardised dilution (1 out of 60), and incubated for 30 min at room temperature. A standard avidin–biotin–peroxidase complex technique was used for visualisation, with diaminobenzidine as chromogen (UltraVision Detection System Anti-Polyvalent, HRP/DAB Ready-To-Use, LabVision Corporation, Fremont, CA, USA). The tissue sections were then lightly counterstained with haematoxylin and cover-slipped.

The following scoring system was used: score 0, no membrane staining or <10% of cells stained; 1+, incomplete membrane staining in >10% of the cells; 2+, weak to moderate complete membrane staining >10% of the cells with; and 3+, strong and complete membrane staining in >10% of the cells. An appropriate positive control (ERBB2-overexpressing breast carcinoma) was included in each run and each section was analysed by a pathologist.

### Fluorescence *in situ* hybridisation

From each gastric adenocarcinoma sample, 4-*μ*m-thick sections of a representative tissue block were cut onto SuperFrost Plus adhesion slides. The slides were then deparaffinised in two series of xylol followed by two series of ethanol (8 min each), rinsed in 2 × SSC, and placed in a solution of NaS/CN 1 M at 80 °C for 10 min. The tissue was then digested with 6 mg ml^−1^ pepsin for 22 min at 37 °C, after which the slides were rinsed in 2 × SSC and dehydrated in an ethanol series. To assess *ERBB2* amplification, a commercial probe (QBiogene, Montreal, Canada now MP Biomedicals, Irvine, CA, USA) targeting *ERBB2*, direct-labelled with rhodamine, and a control probe for chromosome 17 centromere (CEP17), direct-labelled with fluorescein, were used. The slides and probes were placed in a HYBrite denaturation/hybridisation system and co-denatured at 80 °C for 7 min. Hybridisation carried out for 18 h at 37 °C, followed by post-hybridisation washes in 2 × SSC/0.5% Igepal at 73 °C for 5 min and 2 × SSC/0.1% Igepal at room temperature, after which slides were counterstained with DAPI. Fluorescent images were sequentially captured with a Cohu 4900 CCD camera, using an automated filter wheel coupled to a Zeiss Axioplan fluorescence microscope and a CytoVision system.

Gene amplification was scored when a minimum of 60 cancer cell nuclei exhibited a ratio *ERBB2 */ CEP17 ≥2, or when an *ERBB2* signal cluster was observed.

### Statistical analysis

Categorical data were analysed using χ^2^-square test. For parametric data, Student's *t*-test was used when comparing two means. Survival curves were calculated according to the Kaplan–Meier method. Cases lost to follow-up and deaths caused by reasons other than gastric cancer were censored during survival analysis. The significance of differences between survival curves was determined using the log-rank or Breslow's tests. All statistical analyses were conducted using SPSS v.15 (SPSS, Chicago, IL, USA).

## Results

### Overexpression of ERBB2 protein

**Figure 1:**
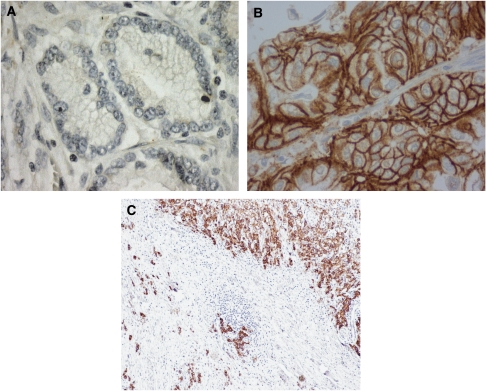
ERBB2 protein expression evaluated by IHC in gastric carcinomas. (**A**) Negative ERBB2 expression (0) (original magnification × 400); (**B**) ERBB2 positive expression –(3+) (original magnification × 400); (**C**) mixed type carcinoma with ERBB2 overexpression in both histological components (original magnification × 100).

The ERBB2 protein status was determined by IHC for the 463 gastric carcinoma tissues (Figure 1). In all, 414 were classified as score 0 (89.4%), 6 were classified as score 1+ (1.3%), 18 were classified as score 2+ (3.9%), and 25 were classified as score 3+ (5.4%).

### *ERBB2* gene amplification

**Figure 2:**
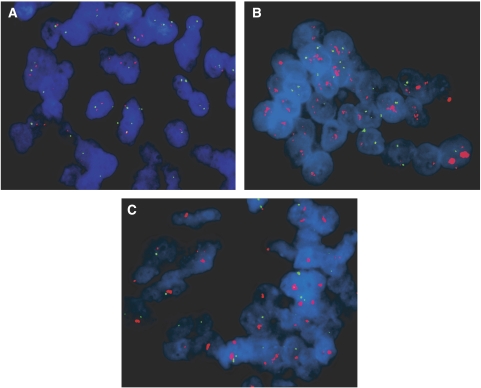
Fluorescence *in situ* hybridisation targeting *ERBB2* in gastric carcinoma specimens (original magnification, × 1000). Red labelled probes target the *ERBB2*, green labelled probes target chromosome 17 centromere, and nuclei (blue) are stained with DAPI. (**A**) intestinal type carcinoma (score 0 by IHC) with no *ERBB2* amplification; (**B**) intestinal type carcinoma (score 2+ by IHC) showing *ERBB2* amplification; (**C**) intestinal type carcinoma (score 1+ by IHC) showing *ERBB2* amplification. A full colour version of this figure is available at the *British Journal of Cancer* online.

Fluorescence *in situ* hybridisation analysis was performed in all cases (*n*=43) in which IHC showed complete membrane immunostaining (2+ and 3+). In addition, 25 cases (including all six 1+ cases) that were regarded as negative for ERBB2 overexpression by IHC were also analysed by FISH. Gene amplification was detected in 38 gastric carcinomas (Figure 2). Only one of the 25 tumours showing negative immunostaining (scored as +1) exhibited *ERBB2* amplification (Figure 2). On the other hand, all 25 cases showing strong complete membrane immunostaining (3+) exhibited *ERBB2* amplification. Among the 18 tumours with 2+ immunostaining, 12 (66.6%) showed amplification (Figure 2). Of the remaining 2+ cases, four showed chromosome 17 polysomy and two showed no genetic alteration.

### Correlation between *ERBB2* amplification and clinicopathological findings

Table 1 shows the clinicopathological differences observed between gastric carcinomas with or without *ERBB2* amplification. Most gastric carcinomas showing *ERBB2* amplification were of the intestinal/glandular type (81.6% of all positive cases, *P*=0.007), but this genetic alteration was also observed in diffuse/isolated cells, and solid and mixed carcinomas (7.9, 5.3, and 5.3% of positive cases, respectively). Two mixed carcinomas showed *ERBB2* amplification and overexpression in the two histological components (Figure 1). *ERBB2* amplification was also associated with an expansive growth pattern (*P*=0.021). Venous invasion, assessed through orcein staining, was not associated with *ERBB2* amplification. No differences were observed between *ERBB2*-amplified and *ERBB2* non-amplified cases in terms of age, gender, type of surgery, and clinical stage.

### Survival analysis

**Figure 3:**
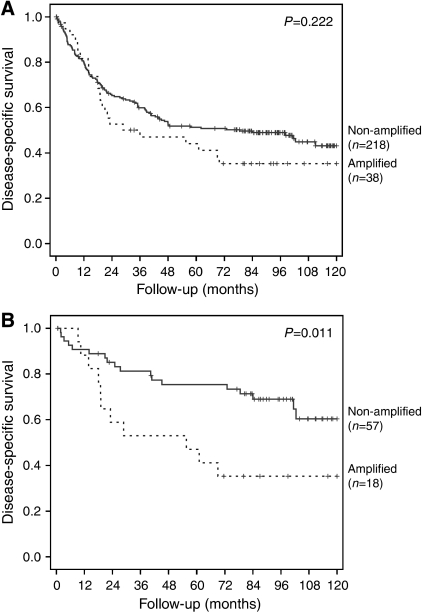
Survival curves of gastric cancer patients, (**A**) Kaplan–Meier plot for disease-specific survival of 256 gastric cancer patients according to *ERBB2* amplification status. (**B**) Kaplan–Meier plot for disease-specific survival of 75 expansive-type gastric cancer patients according to *ERBB2* amplification status.

Survival analysis was performed on 256 patients, including all 38 who showed *ERBB2* amplification. Patients with *ERBB2* amplification had in general, worse 10-year survival rates than those without this genetic alteration (35.3 *vs* 43.2%, respectively; Figure 3), although the difference was not statistically significant (*P*=0.222).

Differences in survival were more evident when we compared similar subgroups of patients. Patients with expansive gastric carcinoma and *ERBB2* amplification had a statistically significant worse survival than those without this genetic alteration (Figure 3; *P*=0.011). No such difference was seen in patients with infiltrative gastric carcinoma (*P*=0.863). Among patients with no lymph node metastases, those with *ERBB2*-amplified carcinomas had a trend for worse survival when compared with those without this genetic alteration (*P*=0.085).

## Discussion

The assessment of *ERBB2* status is essential for efficient selection of patients who might benefit from targeted therapy with trastuzumab or other drugs targeting this surface receptor. This therapeutic option proved useful in extending the survival of breast cancer patients, particularly when selected by FISH (Mass *et al*, 2005). Since then, interest in applying this approach to other malignancies with *ERBB2* amplification has increased. The first goal of this study was to clarify the frequency of *ERBB2* overexpression and amplification in a large series of gastric carcinoma patients (*n*=463). The patients in whom *ERBB2* amplification was found (*n*=38), as well as 218 *ERBB2*-negative patients, were selected for survival analysis. Using clinicopathological and follow-up data from these 256 patients, we investigated whether *ERBB2* amplification is a prognosis factor in different subgroups of gastric cancer patients. Given the nature of the biological material used in this study (formalin-fixed, paraffin-embedded archival tissue), the techniques chosen to assess ERBB2 protein overexpression (IHC) and *ERBB2* amplification (FISH) are the most appropriate to ensure reliable and reproducible results. Moreover, both techniques allow for the specific detection of ERBB2 alteration in individual cells, while maintaining critical architectural tissue information (Pauletti *et al*, 2000).

Both overexpression (9.3%) and amplification (8.2%) frequencies observed in the current study are within the range reported in earlier reports. There was a perfect correlation between IHC and FISH findings in cases with strong (3+) complete membrane staining (100%), as well as in those scored as 0. Kim *et al* (2007) reported 22.6% of carcinomas overexpressing ERBB2 protein (2+ or 3+), but only 7.7% showed gene amplification. As in our study, the correlation was higher in cases with strong (3+) membrane staining and in those scored as 0. Takehana *et al* (2002) also found *ERBB2* amplification in all gastric carcinoma cases scored as 3+. On the other hand, Park *et al* (2006) found *ERBB2* amplification in only 45% of the carcinomas scored as 3+, which might be due to the low specificity of the antibody used. In this study, one of the six gastric carcinomas classified as 1+ showed *ERBB2* amplification. Similarly, Kim *et al* (2007) found *ERBB2* amplification in 4% of the 1+ gastric carcinomas analysed by FISH. Most of the studies that reported the absence of gene amplification in carcinomas classified as 1+ did not analyse all 1+ carcinomas (Takehana *et al*, 2002; Park *et al*, 2006). As for the 18 gastric carcinomas classified as 2+ in this study, 12 (66.6%) showed *ERBB2* amplification and four of the remaining six presented chromosome 17 polisomy. The correlation between 2+ carcinomas and *ERBB2* amplification is higher than usually reported in the literature, possibly because of better specificity of the antibody. In the light of our observations, as well as data from the literature, we recommend that all cases classified by IHC as 1+ and 2+ should be analysed by FISH to avoid false negatives and false positives, respectively.

Some genetic alterations are exclusive of given subtypes of gastric carcinomas, as exemplified by *CDH1* mutations in diffuse gastric carcinomas (Becker *et al*, 1994; Grady *et al*, 2000). Several authors have earlier stated that *ERBB2* amplification is an exclusive event of intestinal-type gastric carcinomas (Takehana *et al*, 2002; Varis *et al*, 2004). However, of the 38 carcinomas with amplification detected by FISH, three were diffuse and four were not classifiable by Lauren. Other authors reported that diffuse gastric carcinomas account for 5–6.2% of all *ERBB2*-amplified carcinomas (Tanner *et al*, 2005; Park *et al*, 2006; Kim *et al*, 2007), which is corroborated by our findings. Interestingly, in both Western and Eastern patients there is a correlation between gastric carcinomas with *ERBB2* amplification and intestinal type: 81.6% in this study *vs* 86 and 84.2% reported in Eastern patients (Park *et al*, 2006; Kim *et al*, 2007).

In two mixed gastric carcinomas containing both isolated cells and glandular components (Carneiro, 1997), *ERBB2* overexpression and amplification were found in both histological patterns. These data indicate that the two histological counterparts of a carcinoma have the same clonal origin and that *ERBB2* amplification is an early genetic alteration acquired before other genetic and/or epigenetic alterations associated with the phenotypic divergence in these cases. This hypothesis is supported by Carvalho *et al* (2006), who studied by array-comparative genome hybridisation 12 mixed carcinomas. Besides finding no significant differences between different histological components of the same tumour, one of the mixed gastric carcinomas studied presented *ERBB2* amplification in both histological counterparts. Furthermore, in our consecutive series of gastric cancer patients, we found *ERBB2* amplification in two early gastric carcinomas (stage IA), as reported earlier in similar studies (David *et al*, 1992; Ooi *et al*, 1998; Park *et al*, 2006; Kim *et al*, 2007), further supporting the idea that *ERBB2* amplification may occur at an early stage in gastric carcinogenesis.

Our findings indicate that the influence of *ERBB2* amplification on patient survival may differ among histological types and pathological staging. The overall survival analysis showed little difference in prognosis according to *ERBB2* status (*P*=0.222), which could be explained by the patient heterogeneity regarding histological and clinical characteristics. According to Ming (1977), expansive carcinomas grow by enlargement of cohesive tumour nodules or masses, with a well-defined tumour boundary. On the other hand, infiltrative carcinomas grow as dispersed, isolated cells, and clusters or small glands that show a strong invasive potential with extensive infiltration into the stroma. This infiltrative characteristic might explain why some authors found that this type of growth pattern was associated with worse prognosis (Davessar *et al*, 1990; Cimerman *et al*, 1994; Luebke *et al*, 2005). Our survival analysis showed that *ERBB2*-amplifying expansive carcinomas have worse prognosis than those with the same growth pattern lacking that genetic alteration (*P*=0.011), which we did not observe in infiltrating gastric carcinomas (*P*=0.863). Recent data published by Wolf-Yadlin *et al* (2006) might explain the different effect of *ERBB2* amplification in carcinomas with different growth patterns. These authors suggested that ERBB2 overexpression promotes increased cell migration but has minimal effect on cell proliferation in cells stimulated by epidermal growth factor or heregulin (Wolf-Yadlin *et al*, 2006). Thus, *ERBB2* amplification could increase cell migration in expansive carcinomas, whereas infiltrative carcinomas, which already have a strong invasive potential, do not acquire additional advantage from *ERBB2* amplification. A similar reasoning may explain our observation of a trend to worse 10-year survival among *ERBB2*-amplified node-negative gastric carcinomas, something that was also reported by others in breast carcinomas (Pauletti *et al*, 2000).

By studying the largest series of gastric cancer patients so far for *ERBB2* amplification and overexpression, we conclude that ERBB2 status may have clinical significance in subsets of patients and that further studies are warranted to evaluate whether gastric cancer patients whose tumours present *ERBB2* amplification/overexpression may benefit from the therapy targeting this surface receptor.

## Figures and Tables

**Table 1:** Comparison of clinicopathological findings between FISH negative and positive gastric carcinomas

	***ERBB2* amplification**		
	**Negative (*n*=218)**	**Positive (*n*=38)**	***P* value**
Age (years)	66 (±10.8)	63 (±13.6)	NS
			NS
*Sex*			
Male	127 (58.3%)	18 (47.4%)	
Female	91 (41.7%)	20 (52.6%)	
			
*Histopathological classification(s)*			
Lauren			0.007
Intestinal	121 (55.5%)	31 (81.6%)	
Diffuse	63 (28.9%)	3 (7.9%)	
Non classified	34 (15.6%)	4 (10.5%)	
Carneiro			0.013
Glandular	119 (54.5%)	31 (81.6%)	
Isolated cells	62 (28.4%)	3 (7.9%)	
Solid	5 (2.3%)	2 (5.3%)	
Mixed	28 (12.8%)	2 (5.3%)	
Non classified	4 (1.8%)		
Ming			0.021
Expansive	56 (26%)	18 (47.4%)	
Infiltrative	153 (71.2%)	20 (52.6%)	
Mixed	6 (2.8%)	—	
			
*Venous invasion*			NS
Positive	147 (68%)	24 (63.2%)	
Negative	69 (32%)	14 (36.8%)	
			
*Nodal metastases* a			NS
N0	73 (34%)	9 (23.7%)	
N+	142 (66%)	29 (76.3%)	
			
*Clinical stage* a			NS
0–IA	26 (12.1%)	2 (5.3%)	
IB	21 (9.8%)	3 (7.9%)	
II	41 (19%)	7 (18.4%)	
IIIA	50 (23.3%)	10 (26.3%)	
IIIB	35 (16.3%)	7 (18.4%)	
IV	42 (19.5%)	9 (23.7%)	
			
*Type of surgery* a			NS
Curative	141 (72.3%)	23 (67.6%)	
Palliative	54 (27.7%)	11 (32.4%)	

NS=non significant.

a Missing cases due to lack of clinical information.
